# The role of remote sensing in tropical grassland nutrient estimation: a review

**DOI:** 10.1007/s10661-023-11562-6

**Published:** 2023-07-15

**Authors:** Adeola M. Arogoundade, Onisimo Mutanga, John Odindi, Rowan Naicker

**Affiliations:** Discipline of Geography, School of Agricultural, Earth and Environmental Sciences, Department of Geography, University of KwaZulu-Natal, Pietermaritzburg, South Africa

**Keywords:** Rangeland, Carbon and nitrogen ratio, Remote sensing

## Abstract

The carbon (*C*) and nitrogen (*N*) ratio is a key indicator of nutrient utilization and limitations in rangelands. To understand the distribution of herbivores and grazing patterns, information on grass quality and quantity is important. In heterogeneous environments, remote sensing offers a timely, economical, and effective method for assessing foliar biochemical ratios at varying spatial and temporal scales. Hence, this study provides a synopsis of the advancement in remote sensing technology, limitations, and emerging opportunities in mapping the *C*:*N* ratio in rangelands. Specifically, the paper focuses on multispectral and hyperspectral sensors and investigates their properties, absorption features, empirical and physical methods, and algorithms in predicting the *C*:*N* ratio in grasslands. Literature shows that the determination of the *C*:*N* ratio in grasslands is not in line with developments in remote sensing technologies. Thus, the use of advanced and freely available sensors with improved spectral and spatial properties such as Sentinel 2 and Landsat 8/9 with sophisticated algorithms may provide new opportunities to estimate *C*:*N* ratio in grasslands at regional scales, especially in developing countries. Spectral bands in the near-infrared, shortwave infrared, red, and red edge were identified to predict the *C*:*N* ratio in plants. New indices developed from recent multispectral satellite imagery, for example, Sentinel 2 aided by cutting-edge algorithms, can improve the estimation of foliar biochemical ratios. Therefore, this study recommends that future research should adopt new satellite technologies with recent development in machine learning algorithms for improved mapping of the *C*:*N* ratio in grasslands.

## Introduction

Globally, grasslands are the second-largest carbon sinks (Latham et al., 2014), accounting for over 40% of the landmass (Bar-On et al., 2018). Grasslands contribute significantly to food security by providing fodder to animals, thus providing livelihoods and economic support for rural communities as well as an array of ecosystem services (Bardgett et al., 2021; Teixeira et al., 2018). These ecosystem services include sequestration of carbon for climate change mitigation, control of soil erosion, and regulation of nutrient cycling (Yuchun et al., 2011; Zhao et al., 2020). The social and societal benefits of grasslands include open spaces for leisure and recreation, cultural practices, and landscape esthetics (Cornelis & Hermy, 2004; Williams, 2015).

Despite their value, grasslands are constantly being degraded at accelerating rates (Bardgett et al., 2021; Muller et al., 2021). The transformation and degradation of grasslands can be attributed to a range of drivers that include climatic variability, invasive plant species, injudicious land use and management, and ecosystem fragility (Li et al., 2022; Tiscornia et al., 2019). The degradation of grasslands leads to a reduction in forage quality and quantity at varying spatial extents (Hooper et al., 2012; Li et al., 2017), thereby adversely affecting livestock production and food security, especially within rangelands. Also, the literature shows that foliar nutrient status/quality in rangelands is regulated by environmental conditions such as changes in temperature, precipitation, and soil nutrients, causing spatial variation in rangelands (Getabalew & Alemneh, 2019; Ghorbani et al., 2020). Therefore, a good understanding of rangeland properties is fundamental to developing robust analytical frameworks that can be used to obtain insightful information on their conditions and inform various strategic and operational decisions. Hence, rangeland managers must monitor grassland health/nutrient utilization to understand their effect on grassland productivity and quality.

Foliar biochemicals/nutrients play a major role in forage health and quantity. Foliar nutrients are an important variable in determining plant physiological status because it is linked to biomass productivity and vegetation health (Mokgakane, 2021). Foliar nutrients including nitrogen (N) and carbon (C) are important indicators of the quality and quantity of grasslands. Foliar N is important to vegetation because it constitutes amino acids, an important building block of proteins, as well as nucleic acids, chlorophyll, and other cellular components (Mu & Chen, 2021). In plants, the amount of nitrogen determines their growth, vigor, developmental stage, and functional role (Band et al., 2022; Leghari et al., 2016). Studies such as Yang et al. (2022) and Mu and Chen (2021) have highlighted that plants utilize and require N more than other nutrients. This is largely reflected in the large amount of N used during photosynthesis, especially Rubisco and molecules responsible for harnessing light energy (Evans & Clarke, 2019; Nunes-Nesi et al., 2010). The quantity of N invested enables the conversion of carbon dioxide, water, and inorganic nitrogen to make sugars, organic acids, and amino acids, which are essential for biomass production (Nunes-Nesi et al., 2010). Therefore, the availability of N determines both photosynthetic capacity and plant production (Nasar et al., 2021). In addition, the bond between C and N pathways is essential to the growth and development of plants. This is because photosynthesis requires a large amount of N, so, for optimal CO_2_ absorption through photosynthesis and consequently biomass production, enough N supply is required (Mengesha, 2021). In summary, the amount and distribution of N are key components of the carbon cycle and are valuable indicators of plants’ metabolism and development. According to Kocheva et al. (2020), the concentration of leaf N is related to plant photosynthetic capability, plant primary production, respiration, and the productivity and sequestration of C (Tang et al., 2018). The ecosystem C storage is limited due to N availability, affecting among others litter decomposition, soil organic carbon, and the allocation of C to various plant organs.

Grassland nutrients are affected by abiotic and biotic factors, including topography, temperature, precipitation, organic matter, and grazing (Mokgakane, 2021; Ravhuhali et al., 2021; Zhang et al., 2021). Changes in the C and N levels in grasslands over time often lead to variations in the carbon-nitrogen (*C*:*N*) ratio (Onandia et al., 2019), thereby affecting the nutrient cycling and biomass in rangelands. The *C*:*N* ratio is critical in plant functioning and growth and is widely adopted within both the field of ecosystem evolution and global climate change studies (He et al., 2006). It is a measure of the efficiency with which nutrients are utilized by plants and influences the redistribution of biomass from root to shoot. Also, it promotes the accumulation of soil organic carbon, regulation of litter decomposition in plants, photosynthesis, and net primary production and to an extent indicates the growth rate of plants (Grechi et al., 2007). Within rangelands, grazing animals require vegetation with high protein and a low *C*:*N* ratio <36 (< 36g of carbon for each 1g of nitrogen) (Beeri et al., 2007). This information is useful in rangeland management to determine when pasture cannot meet the minimum animal maintenance requirements. Despite grasslands’ economic importance and their role in C and N cycling, there is a lack of spatially explicit data on grassland biophysical and biochemical properties. Therefore, to adequately monitor nutrient dynamics and productivity, a better understanding of the *C*:*N* ratio fluctuations in rangelands is needed (Liu et al., 2021). However, no study has been undertaken to synthesize the available literature on the importance of the *C*:*N* ratio within rangelands. This will be beneficial to decision-makers in rangeland management.

The traditional methods used in the determination of foliar N and C are based on plant measurements and laboratory analysis (Catchpole & Wheeler, 1992; Sáez-Plaza et al., 2013). The estimation of foliar nitrogen has been done through laboratory techniques using the Kjeldahl digestion technique (Sáez-Plaza et al., 2013) and Duma’s combustion methods (Simonne et al., 1997). However, these methods are laborious, require long processing time, and are costly; spatially restricted and chemical reagents used during analysis can destroy the samples (Muñoz-Huerta et al., 2013), while carbon has been estimated based on the biomass information derived through the direct/destructive method. The direct (plant-based) methods for estimating grassland above-ground biomass (AGB)/carbon stocks are based on plots and allometric equations from *in situ* measurements (Catchpole & Wheeler, 1992). The direct method involves clipping and weighing grass samples in the field for further laboratory analyses (Schaefer, 2015). While this approach is regarded to be accurate, it is not ideal for repeated measurements, time-consuming, laborious, and expensive. Furthermore, leaf sampling and analysis are susceptible to human error, leading to inconsistencies and bias that may result in compromised data interpretation and significance. The direct estimation of foliar carbon and nitrogen individually may result in a *C*:*N* ratio that is susceptible to error, due to the inconsistent orders in the magnitude of C and N (Gao et al., 2020). Thus, assessing the foliar *C*:*N* ratio directly with high-resolution sensors may aid in reducing these errors. This is due to the high spectral and spatial resolutions that enable discrimination and mapping of features with relatively similar properties, with associated minimized errors (Gao et al., 2020; Reddy, 2021). Using hyperspectral data, Gao et al. (2020) reported that red and red edge bands are sensitive to the retrieval of *C*:*N* ratio in rangelands. In another study, Phillips et al. (2006) estimated canopy *C*:*N* ratio in a mixed-grass prairie field with <14% and 9.6 % error using Landsat 5 and ASTER data, respectively.

Remote sensing offers a better alternative approach to understanding the estimation of rangeland foliar nutrient ratio in an ecosystem at several spatial and temporal resolutions (Ramoelo et al., 2012; Shen et al., 2020). Remotely sensed data offers a timely, economical, and efficient approach to monitoring rangelands’ foliar biochemicals for assessment and management (Lu et al., 2020; Ramoelo et al., 2012). In addition, present advancements in sensor technology have enhanced the estimation and detection of changes in grassland health and biomass (Schucknecht et al., 2021; Zhao et al., 2021). In addition, remotely sensed data can be integrated with ancillary data, augmenting data-based decision-making in rangeland management.

Despite this knowledge, very few studies have utilized remote sensing to map the *C*:*N* ratio in grasslands. As a result, a review of techniques used within grasslands is therefore required along with areas and gaps that need further research. This will be important to scientifically identify the priorities and challenges for future research in the application of remotely sensed data in rangeland studies. Previous reviews have focused majorly on the remote sensing of either foliar carbon or nitrogen in forests, crops, and rangelands. For instance, Xiao et al. (2019) reviewed the developments in remote sensing platforms and sensors of the carbon cycle over 50 years (1970 to 2019), while Naicker et al. (2019) did a quantitative review of foliar nitrogen using remote sensing. In a related study, Wei and He (2020) conducted a global systematic review of the foliar *C*:*N* ratio in urban trees. Generally, there has been little research on the adoption of remote sensing techniques in quantifying the foliar *C*:*N* ratio, which is important in rangeland management. As such, this study examined remote sensing’s opportunities to estimate foliar *C*:*N* ratios in rangelands, as well as its challenges and prospects. Such knowledge is important due to recent advancements in remote sensing technology. For example, recent advancements in broadband multispectral sensors (Sentinel 2 and Landsat 8/9) with improved spectral and spatio-temporal resolutions provide new options to estimate foliar biochemical ratios in grasslands. Hence, this study provides a synopsis of remote sensing techniques to determine the foliar *C*:*N* ratio in grasslands and their associated challenges, opportunities, and prospects. Our study (1) will focus on the remote sensing of foliar *C*:*N* ratio, (2) analyze the various statistical and empirical methods used in the estimation of foliar *C*:*N* ratio, and (3) highlight the challenges and future research necessary to estimate foliar *C*:*N* ratio.

## Remote sensing of foliar carbon to nitrogen ratio

The earliest effort to demonstrate the relationship between carbon and nitrogen in vegetation was by Blackman (1919), using laboratory-based techniques. Blackman (1919) developed Blackman’s concept, which reports an increase in carbon levels during catalytic activity in plants. During plant growth, the carbon-nitrogen interaction model can be developed using Equation (1) below.

1 $$DM= DM0{e}^{CN1\ast N}$$

where DM is the final dry weight of the plant at a particular time, nitrogen (N)% is the amount of nitrogen stored in the plant at a particular time, DM_0_ is the initial dry weight, and CNI is the carbon-nitrogen index, which is the value for ΔDM/ΔN/DM at a particular given period.

Blackman (1919) found that the productivity of plants is determined by their carbon and nitrogen content since plants’ nitrogen content and their photosynthetic rates are closely related. Increasing photosynthesis results in high nitrogen uptake by roots through enhanced water flow and root activity because carbohydrates are distributed widely throughout the roots. Therefore, it is presumed that there is a mutual regulation between carbon and nitrogen in plants.

As a result of this relationship, scientists have explored the relationship between C and N in plants using field and laboratory techniques (Corbesier et al., 2002; Melillo et al., 1989; Osaki et al., 1992; Shinano et al., 1991; Tanaka & Osaki, 1983). Although these approaches are accurate, they are expensive, laborious, and not practical at regional scales or remote areas. Therefore, as aforementioned, remote sensing is a cheaper and more spatially oriented alternative in the mapping and monitoring of foliar *C*:*N* ratio in plants due to its repetitive acquisition of spectral information at both local and regional scales. A selected number of researchers have demonstrated the capability of optical remote sensing data with machine learning algorithms and the radiative transfer method to detect the *C*:*N* ratio in plants (Féret et al., 2021; Gao et al., 2020; Wei & He, 2020; Xu et al., 2018). However, remote sensing applications in *C*:*N* ratio estimation at a large scale and in heterogenous environments is limited (Gao et al., 2020). Therefore, we explore the utility of remote sensing technology in *C*:*N* ratio estimation at various scales through an extensive review of the literature.

## Hyperspectral remote sensing of foliar *C*:*N*

Hyperspectral sensors, also known as imaging spectroscopy, contain many narrow continuous bands through the visible, near-infrared, and short wave infrared regions of the electromagnetic spectrum (Berger et al., 2020; Mutanga et al., 2003). Hyperspectral data can better distinguish and estimate subtle biochemical and biophysical properties in grasslands (Marabel & Alvarez-Taboada, 2013; Schweiger et al., 2015; Yu et al., 2020) compared to traditional broadband (> 100 nm) multispectral data (Kumar et al., 2001), due to their several bands. Hyperspectral data are primarily collected by handheld spectrometers or airborne sensors. Since the invention of the airborne visible/infrared imaging spectrometer (AVIRIS) in 1987, hyperspectral sensors have been utilized in vegetation study. For example, using AVIRIS data, Kupiec and Curran (1995) examined whether the canopy effect (structure, biomass, LAI, and shadow) altered foliar nutrient concentrations in the canopy reflectance spectral region. The study demonstrated that at wavelengths beyond 1400nm, the canopy influences leaf reflectance, while near-infrared leaf properties remain unchanged.

Several studies (Beeri et al., 2007; Chen et al., 2019; Gao et al., 2020; Lihong et al., 2006; Xu et al., 2018) have explored the capabilities of hyperspectral data in determining the foliar C: N ratio in vegetation. According to Xu et al. (2018), the monitoring of foliar *C*:*N* ratio using remotely sensed data, especially with hyperspectral imagery, is still at the exploration stage. Using two sets of field data with different nitrogen levels and rice cultivars, Zhou et al. (2009) suggested that band 672nm could monitor foliar *C*:*N* ratio in rice using canopy hyperspectral parameters. Also, Lihong et al. (2006) established that NDVI derived from the integration of two spectral bands at 710nm and 1650nm from rice canopy reflectance spectra could map leaf *C*:*N* ratio. Building on this, Xu et al. (2018) investigated the use of spectral response curve from canopy hyperspectral reflectance data with the Branch and Bound algorithm to retrieve foliar *C*:*N* ratio in wheat and barley. Their results indicated the change in the *C*:*N* ratio could be evaluated with an accuracy of *R*^2^ of 0.63, 0.68, and 0.65 for wheat, barley, and both species combined respectively, using the best slope feature. Another attempt to map the *C*:*N* ratio was by Gao et al. (2020) in the alpine grasslands, China, using field hyperspectral data and random forest algorithm. The authors found the red and red edge bands to be useful in estimating *C*:*N* ratio using the random forest algorithm.

However, despite the accuracy obtained from these sensors, data acquisitions are often hampered by plants’ canopy characteristics (leaf area index and canopy cover) (Jiang et al., 2021). Gara et al. (2018) demonstrated that variation in light availability and leaf shading affects the biochemical and morphological characteristics including chlorophyll, nitrogen, dry weight, and photosynthesis due to different canopy structures. These often result in different spectral values between the top and the bottom surface of the same leaf. For instance, Chen et al. (2019) quantified the leaf carbon, nitrogen, and *C*:*N* ratio in soya beans under different light conditions using hyperspectral reflectance. The results showed that leaf nitrogen increased while carbon decreased with an increase in shading. They concluded that the continuous wavelet transformation model had the lowest root mean square error (RMSE) of 1.9789, 0.6132, and 2.1587 for carbon, nitrogen, and *C*:*N* ratio, respectively. However, whereas these studies have demonstrated that light variation/shading influences the plant biochemical contents, there has been a lack of focus on quantifying the effect of different shading levels/light variations on *C*:*N* ratio in plants, especially within rangelands.

Some studies (Chen et al., 2019; Gao et al., 2020; Xu et al., 2018) indicate that near-infrared and red edge bands can map *C*:*N* ratio in vegetation. These studies have demonstrated the effectiveness and capability of hyperspectral sensors to retrieve foliar *C*:*N* ratio in grasslands. Results from the aforementioned studies indicate the possibility of estimating rangelands’ *C*:*N* ratio using hyperspectral sensors’ spectral properties. In summary, hyperspectral data can capture the subtle variation in vegetation due to hundreds of bands ranging from 350nm to 2500nm, which is critical for plant monitoring. However, hyperspectral data is costly, especially for regional mapping, and not readily available (Feifei et al., 2020; Tong et al., 2013). Furthermore, the absorption features of most foliar biochemicals, such as cellulose, lignin, nitrogen, and starch, are affected by water in fresh leaves. Due to this challenge, Gao and Goetz (1994) developed the water removal approach to deal with the masking effect of water from fresh leaves. Following up on the study, Schlerf et al. (2010) modified the technique.

## Multispectral sensors in estimating foliar *C*:*N*

The conventional multispectral sensors including MODIS, SPOT, ASTER, and Landsat series with medium to coarse spatial resolution have been used to estimate plant nutrients. They are usually limited to the regional mapping of vegetation’s physical and chemical properties because of their limited spectral channels and discontinuous bands (Anderson et al., 1993; Yin et al., 2015). The remote sensing fraternity recently witnessed the advancement in technology of freely available optical sensors with improved spectral, radiometric, and spatial resolutions such as Landsat 8/9 and Sentinel 2 suitable for mapping grasslands’ morphological and biochemical properties (Adagbasa & Mukwada, 2022; Pang et al., 2022; Soltanian et al., 2021). Therefore, it is necessary to adopt these freely available multispectral sensors to map foliar nutrient ratios such as *C*:*N* ratio. However, Rahman et al. (2020) noted that there is limited application in assessing leaf *C*:*N* ratio using freely available sensors, including Landsat 8 and Sentinel 2. Rahman et al. (2020) utilized Landsat 8 and Landsat TM5 bands, texture metrics, and indices to map the spatiotemporal variation of *C*:*N* ratio of senescent leaves in a reserved forest using machine learning techniques. Their study reported that bands sensitive to moisture and temperature (thermal and short wave infrared bands) are the top predictor in modeling the foliar *C*:*N* ratio using freely available Landsat TM data. The effectiveness of Landsat 8 can be attributed to the push broom scanner with a high signal to noise ratio that predicts foliar properties more accurately compared to its predecessors. Despite the noticeable success of the study by Rahman et al. (2020), there is a gap in the use of the Landsat series to predict *C*:*N* ratio, particularly in rangelands. So, future research must examine the strength of recent sensors such as Landsat 8/9 with improved spectral and spatial properties in estimating *C*:*N* ratio in grasslands. Notwithstanding, in recent times, there has been a shift toward advanced, cheaper, or freely available multispectral sensors equipped with red edge bands suitable for determining foliar nutrients.

Furthermore, advanced and readily available multispectral sensors, including Sentinel-2, have additional red edge spectral regions, thus comparable to commercial sensor systems (WorldView-3 and RapidEye) with a high spatial resolution (Omer et al., 2017; Sagan et al., 2021; Westergaard-Nielsen et al., 2021). The freely available Sentinel 2 is equipped with 13 absorption wavebands in the visible to shortwave infrared, with additional red edge bands (705, 740, and 783 nm) (Adagbasa & Mukwada, 2022). Mutanga and Skidmore (2007), Clevers and Gitelson (2012), and Koley and Chockalingam (2022) illustrated that the red edge bands are sensitive to the vegetation properties (nitrogen, biomass, and canopy structure). Despite these studies producing reasonable results, few studies have used advanced multispectral sensors to estimate *C*:*N* ratios. For example, Westergaard-Nielsen et al. (2021) quantified the spatiotemporal variations in Artic tundra leaf *C*:*N* ratio based on the new Sentinel 2-derived index. The results showed that the normalized reflectance index (NRI_1610_) derived from the shortwave infrared and red edge bands, estimated the *C*:*N* ratio with an accuracy of *R*^2^ = 0.81. From the aforementioned study, the use of advanced and open source multispectral sensors based on the red-edge spectral bands have the potential to determine the *C*:*N* ratio of rangelands. This will augment the available understanding of estimating the rangeland biochemical ratio in future research.

In addition, the integration of sensors in the estimation of nitrogen and biomass may be beneficial in providing additional information for complex applications in heterogeneous areas with varying structural properties. This is because of the unique spatial and spectral resolution in each sensor. However, there are no available studies on the benefits of sensor integration in estimating foliar nutrient ratios such as *C*:*N* ratio, especially in rangelands. In recent times, the fusion of optical, LIDAR, and radar data has improved model accuracy for biomass and quality estimation of highly heterogeneous grasslands. For example, Grüner et al. (2020) combined terrestrial laser scanning (TLS) with unmanned aerial vehicle-based multispectral (MS) data to estimate biomass and nitrogen fixation in different grass-legume mixtures. The fusion of TLS and MS yielded the best accuracy with a relative root mean squared error of prediction (rRMSEP) of 14%, while MS (rRMSEP of 18%) and TLS (rRMSEP of 21%) for nitrogen fixation in the grass-legume mixture. The study highlights the importance of point cloud data and optical sensors in improving model prediction in vegetation studies, which can be applied in the estimation of foliar *C*:*N* ratio. Therefore, this integration of sensors may be beneficial in improving the prediction of the *C*:*N* ratio in rangelands.

Presently, cost-effective unmanned aerial vehicles (UAVs) are developing technologies in the estimation of grasslands parameters. In addition to providing high-spatial-resolution imagery, UAVs are also less subject to cloud and haze interference, making them suitable for measuring grassland aboveground biomass and biochemicals (Franceschini et al., 2022; Schucknecht et al., 2022). For instance, Schucknecht et al. (2022) evaluated the effectiveness of UAV-borne multispectral data for determining dry biomass and nitrogen (N) concentration of pre-Alpine grasslands. The authors produced a relative root mean square error (average cross-validated) rRMSE_cv_ of 12.6 % for dry biomass and rRMSE_cv_ of 14.2 % for N model using the raw reflectance and vegetation indices. It is therefore hypothesized that the robustness of UAVs might improve the mapping of foliar biochemical ratios in grasslands. Future research should test the strength of UAVs in monitoring grasslands’ *C*:*N* ratio.

## Influential spectral variables in estimating *C*:*N* ratio

The estimation of foliar nutrients depends on absorption features in the near-infrared and shortwave infrared region (Knox et al., 2012). Studies such as (Curran et al., 2001) and (Mutanga & Skidmore, 2004a) have identified absorption features related to biochemical nutrients in vegetation. For instance, the absorption bands (1020nm, 1510nm, 1730nm, 1980nm, 2060nm, 2130nm, 2180nm, 2240nm, and 2300nm) have been used to quantify nitrogen, chlorophyll, and protein of foliar biochemical (Curran, 1989; Gao et al., 2020; Naicker et al., 2019; Ramoelo et al., 2013). Similarly, some known absorption bands (910 nm, 930 nm, 1020 nm; 1040 nm, 1120 nm, 1510 nm, 1690 nm, 1730 nm, 1780 nm, 1980 nm, 2000 nm, 2060 nm, 2100 nm, 2130 nm, 2180 nm, 2240 nm, 2270 nm, 2280 nm, and 2300 nm) of cellulose, starch, lignin, and sugar absorbing in the SWIR region have been used in the detection of carbon compounds in vegetation (Gao et al., 2020; Pullanagari et al., 2012; Thulin et al., 2014), since specific wavebands for carbon have not been identified.

Also, parameters in the red and red edge region (slope, position, amplitude, and index) have produced promising results in predicting grasslands N, chlorophyll, cellulose, lignin, carbohydrates, and biomass content (Gao et al., 2020; Guerini Filho et al., 2020; Mutanga & Skidmore, 2007). For example, Durante et al. (2014) identified absorption bands in the red, red-edge, and SWIR regions that could predict the *C*:*N* ratio in grass using leaf spectral reflectance. In barley and wheat leaves, Xu et al. (2018) noted that based on the spectral slope features, bands in the red edge performed better at predicting the *C*:*N* ratios. In another study, Gao et al. (2020) tested the strength of hyperspectral data bands to determine the *C*:*N* ratio in grasslands using the random forest and support vector machine algorithms. According to their findings, the red, red edge, and SWIR (1950–2350 nm) bands performed well in predicting foliar *C*:*N* ratios, with coefficients of determination of validation (*V*-*R*^2^) ranging from 0.70 to 0.80.

The empirical-based model of estimating foliar biochemicals involves the use of vegetation indices, absorption features, full-spectrum, and integrated modeling. The estimation of foliar biochemicals has been extensively done using vegetation indices, which combine different spectral reflectance bands. Traditional broadband indices (Normalized Difference Vegetation Index, Modified Simple Ratio, and Soil Adjusted Vegetation Index) have been explored to quantify the biochemical content of plants (Farella et al., 2022; Rahman et al., 2020). In particular, Gao et al. (2020), Rahman et al. (2020), and Lihong et al. (2006) utilized spectral indices from traditional broadband indices (NDVI, SAVI) in the prediction of the *C*:*N* ratio. Lihong et al. (2006) reported that NDVI with bands 710nm and 1650nm could determine the *C*:*N* ratio of rice at the late growth stage. Also, Rahman et al. (2020) ranked Landsat TM5 and Landsat 8 vegetation indices among the top 10 predictors in mapping the spatiotemporal variations of *C*:*N* ratio of senescent leaves in a reserved forest, Bangladesh. However, these conventional broadband indices suffer from saturation in dense vegetation and are insensitive to subtle changes in foliar biochemicals (Mutanga & Skidmore, 2004b).

In contrast, several researchers have concluded that red-edge indices of hyperspectral data and advanced multispectral data can reduce the saturation effects of traditional broadband indices (Imran et al., 2020; Liu et al., 2022; Ramoelo et al., 2015). In leaf, the red-edge region is the rapid rise in reflectance between 680nm and 780nm (Mutanga & Skidmore, 2007). In addition, the red-edge bands from advanced broadband sensors such as Sentinel 2, World view, and Rapid Eye have been reported to improve the prediction accuracy of foliar nutrients (Imran et al., 2020; Ramoelo et al., 2012; Vasudeva et al., 2021). For instance, Vasudeva et al. (2021) mapped the spatial distribution of forest nitrogen and carbon in India using Sentinel 2 band and vegetation indices and machine learning algorithms. Their investigation showed that the random forest algorithm could accurately predict foliar nitrogen and carbon with an *R*^2^ of 0.85 and *R*^2^ of 0.86 and the most important indices in predicting C and N had red edge bands. The success of Sentinel 2 in estimating foliar nutrients (carbon and nitrogen) is due to the strategically positioned red edge bands, the 10 to 60m spatial resolution, and the high temporal resolution (5 days) which is adequate for monitoring and management of rangelands (Imran et al., 2020). Although the red edge in Sentinel 2 MSI has been used extensively in estimating nitrogen in vegetation, the effectiveness of Sentinel 2 data to estimate foliar carbon is at an exploratory stage, and a clear understanding of the spectral properties that can estimate carbon in plants is necessary. Furthermore, the strength of new red edge indices (red edge normalized difference vegetation indices, inverted red-edge chlorophyll index, and Sentinel 2 red edge position index) have been documented to have high predictions in estimating foliar nutrients (Koley & Chockalingam, 2022; Liu et al., 2022). There is a need to test these new red edge indices in mapping the *C*:*N* in rangelands, as no such literature exists. Hence, it can be concluded that the spectral properties of plants either from field measurements, UAVs, airborne sensors, or satellite imagery influence the effectiveness of vegetation indices based on foliar ratios such as the *C*:*N* ratio. However, Pacheco-Labrador et al. (2014) also noted that vegetation indices could be affected by differences in plant type, season, and the impact of the range in canopy foliar concentration.

In summary, the literature notes that absorption features sensitive to *C*:*N* ratio dominates the near-infrared, SWIR, red, and red edge regions (Beeri et al., 2007; Gao et al., 2020; Rahman et al., 2020). Also, spectral transformation techniques (continuum removal, derivatives) improve the accuracy in predicting *C*:*N* ratio in grasslands (Gao et al., 2020). A large number of the studies on the estimation of *C*:*N* ratio are largely dominated by hyperspectral data using a machine learning algorithm in forests and croplands. This is due to the ability of hyperspectral sensors to detect minute details in vegetation. Also, there is limited research in the application of freely available, advanced multispectral sensors (Sentinel 2 and Landsat 8) in predicting foliar biochemical ratios in rangeland, especially the *C*:*N* ratio. Furthermore, there is a gap in the application of modern remote sensing technology such as UAVs to predict the *C*:*N* ratio in grasslands. The use of UAVs holds better prospects in estimating rangeland *C*:*N* ratio due to its low cost, flexibility in data acquisition and sensor integration, and wide field of view.

## Regression and machine learning algorithms utilized in estimating *C*:*N* ratios

The challenges of analyzing remotely sensed data includes, the processing of a large amount of data, varying spatial-temporal and spectral properties, and a wide range of proximate bands. Therefore, techniques that can analyze, integrate and help make the best-informed decisions from the huge amount of datasets are necessary. Several studies have adopted techniques such as parametric and non-parametric (linear regression, non-linear regression) in mapping foliar nutrients using remotely sensed data (Das et al., 2020; Maimaitijiang et al., 2020; Prado Osco et al., 2019). In particular, the non-parametric models (linear and nonlinear machine learning algorithms) compute coefficients to reduce the error associated with variables extracted. As a consequence, the model development is simplified, since no explicit parametrization is necessary; however, it may require a higher level of expertise to understand and execute these models (Verrelst et al., 2019).

Multivariate linear regression methods such as the stepwise multiple linear regression (SMLR), partial least square regression (PLSR), and support vector machine (SVM) have been used as predictive models to estimate plant nutrients (Berger et al., 2020; Hou et al., 2018). Stepwise multiple linear regression (SMLR) was commonly utilized in earlier studies for the extraction of nutrient content from spectral data (Kokaly, 2001; Mutanga et al., 2004). Peng et al. (2020) used stepwise linear regression and hyperspectral data to map leaf nutritional status in degraded plants with an *R*^2^ = 0.5–0.8 and *p* < 0.05. However, these methods (i.e., SMLR) assume that data is normally distributed and so may suffer from overfitting and multicollinearity, especially in hyperspectral datasets (Huang et al., 2004). In contrast, PLSR produces robust and better models due to its ability to reduce spectral data into fewer orthogonal variables. However, the relationship between plant variables and spectra data is non-linear and complex, so PLSR might not be ideal for hyperspectral analysis. For example, to estimate leaf nitrogen content, Yi et al. (2014) compared SMLR to PLSR and nonlinear machine learning regression algorithms. The nonlinear regression outperformed the SMLR and PLSR due to their flexibility.

Non-linear non-parametric regression, also referred to as machine learning algorithms (ML), has the advantage of capturing nonlinearity among image features without relying on the underlying distribution of data. Machine learning algorithms (i.e., random forest (RF) and Stochastic Gradient Boosting (SGB)) have the potential to rapidly generate adaptive and robust relationships, once trained (Barzin et al., 2021; Chlingaryan et al., 2018). These algorithms have been used to estimate plant biomass and nutrients. Machine learning algorithms can handle the nonlinearity between vegetation parameters (biochemical and biophysical properties) and the reflected radiance (Chlingaryan et al., 2018). They may therefore be more appropriate in vegetation studies. Some studies (Gao et al., 2020; Shi et al., 2021; Xiao et al., 2019) have illustrated that non-linear regressions (stepwise multiple linear regression) and kernel-based extreme learning machine regression (cubist and extreme learning regression) are superior to linear regressions in quantitative models. This is because they are flexible and can handle highly correlated predictors with efficiency in picking the key predictors among several predictors (Prado Osco et al., 2019; Pullanagari et al., 2016).

For example, Pullanagari et al. (2016) utilized airborne hyperspectral data with non-linear machine algorithms (RF, SVM) and linear PLSR in predicting several foliar nutrients in a mixed pasture. Their study indicated that RF and SVR outperformed PLSR in estimating different foliar nutrients. Similarly, Maimaitijiang et al. (2020) and Gao et al. (2020) successfully mapped the plant *C*:*N* ratio with RF. The random forest algorithm has an excellent ability to overcome the issues of multi-collinearity and to investigate the internal relationships of specific foliar biochemical and biophysical properties with multiple spectral data (Fernández-Habas et al., 2022; Otgonbayar et al., 2019). The RF comprises different decision trees that are widely applied in varying classification and regression problems (Maimaitijiang et al., 2020). These machine learning models have the advantage of easily handling several predictor variables derived from ancillary and remotely sensed data related to foliar nutrients and carbon stocks (Xiao et al., 2019). Using the RF approach to predict the C: N ratio in grasslands from *in situ* hyperspectral data has also produced promising results Gao et al. (2020). Shi et al. (2021) concluded that the random forest not only has the advantage over the support vector machine but also exhibits model simplicity and circumvents overfitting.

However, artificial neural networks (ANNs) have multiple variants with various topological structures (Chen et al., 2013; Liu et al., 2018), making them flexible with the ability to generalize and perform efficiently with the appropriate setting of model parameters. ANN comprises multiple hidden and output layers of interconnected groups of nodes (Torkashvand et al., 2020). Each neuron is trained to produce outputs based on some activation function, training algorithm, initial weight, and biases (Wang et al., 2009). However, the ANN suffers from overfitting, which may likely influence model prediction. Different studies have used algorithms such as the “save best” and “early stopping” strategies to prevent overfitting in the ANN (Srivastava et al., 2014).

Another attempt to estimate *C*:*N* ratio using the machine learning algorithm was by Rahman et al. (2020) in the mangrove ecosystem between Bangladesh and India using SGB, RF, SVM, and PLSR. The SGB, RF, and SVM performed better than the PLSR. They concluded that the *C*:*N* ratio and predictor variables are non-linear related in the reserve forest, hence the weak performance of the PLSR modeling technique which is linear based (Sun et al., 2019). The aforementioned studies indicate that non-linear models have better performance in predicting *C*:*N* ratio in plants. Therefore, studies on *C*:*N* ratio in rangelands should focus on the improved accuracy of non-linear models as there is a paucity of data in this regard.

In recent times, Das et al. (2020), and Mahajan et al. (2021) concluded that models which integrate linear with non-linear algorithms can improve model accuracy, better than individual multivariate techniques in the estimation of foliar nutrients (Das et al., 2020; Mahajan et al., 2021). According to Das et al. (2020), the robustness of these integration models lies in their ability to reduce multi-collinearity problems and increase processing speed, while retaining most of the information in the original dataset. Also, the use of principal components, latent variables, and selection of variables through variable importance as input for further machine learning modeling makes this method possible (Mouazen et al., 2010). In addition to reducing collinearity, data dimensionality, and speeding up computation, these approaches preserve most of the original dataset's information (Yang & Ge, 2020). The integration of linear and non-linear regression analysis has been demonstrated in a few studies, but very limited information is available on this aspect in grasslands, specifically mapping foliar *C*:*N* ratio in rangelands. The combination of these models is important to evaluate whether the integration of models will provide robust results in comparison with individual ones in estimating foliar *C*:*N* ratio in grasslands.

Nevertheless, machine learning algorithms are limited as they are designed to suit certain types of data. Consequently, a method that works for one task may not be as effective for another. In addition, the original reflectance spectra require several pre-processing methods (first derivative, continuum-removal) to obtain more accurate predictions when using the multivariate regression methods (Gao et al., 2020; Ramoelo et al., 2013). It is therefore possible to achieve different predictive outcomes by using different spectral pre-processing techniques. Hence, it is challenging to balance between model complexity and accuracy when choosing preprocessing and multivariate regression methods.

Recently, the use of deep learning (DL) algorithms: convolutional neural networks and Stacked Sparse Autoencoder networks that present data hierarchically, has attracted broad attention in vegetation studies (Ahsan et al., 2021; Maimaitijiang et al., 2020). The DL algorithm has been successfully used to map foliar biophysical and biochemical properties (Azimi et al., 2021; Buxbaum et al., 2022). DL is a unique ML algorithm that utilizes several layers of non-linear information to model complex relations among data. As a result of its ability to rely primarily on data for image recognition, deep learning is considered to be a powerful tool (Buxbaum et al., 2022). Furthermore, Pullanagari et al. (2021), Odebiri et al. (2021), and Yuan et al. (2020) have reported the superiority of DL over ML and geostatistical methods. For example, using a large field spectroscopy database, Pullanagari et al. (2021) compared the accuracy of one-dimensional convolutional neural network (1D-CNN) with PLSR and gaussian process regression (GPR) in estimating canopy nitrogen in grassland. The results showed that in comparison to PLSR (0.31) and GPR (0.16), prediction derived using 1D-CNN achieved greater accuracy with <0.12 mean standard deviation. Therefore, due to its optimal accuracy and better performance, it is necessary to evaluate the utility of DL in mapping *C*:*N* ratio in rangelands, as there is no available literature. Despite the optimal performance and higher accuracies of emerging machine learning algorithms in the estimation of foliar nutrients, no standard method has been identified to be optimal for mapping foliar nutrients using different remotely sensed data. Hence, future studies need to investigate the use of more robust algorithms to estimate *C*:*N* ratio in rangelands.

## Radiative transfer model in the estimation foliar *C*:*N* ratio

Radiative transfer models (RTMs) are physical models that depict how solar radiation interacts with vegetation based on optics laws (Myneni et al., 1992). These RTM models include PROSPECT (Jacquemoud & Baret, 1990), PROSAIL (an integration of PROSPECT which is a leaf level model, and SAIL which is a canopy level model), and LIBERTY (Dawson et al., 1998). Earlier studies on the reflectance of leaf modeling were established on the “Kubelka-Munk” theory of radiative transfer (Allen & Richardson, 1968). Jacquemoud and Baret (1990) derived the PROSPECT model from Allen et al. (1969) generalized plate model, which describes leaf optical properties from 400nm to 2500nm. The RTMs have been widely used in predicting foliar biochemical such as dry matter, water, and chlorophyll (Darvishzadeh et al., 2008; Feret et al., 2008; Sun et al., 2019), with limited studies on cellulose, lignin, protein of plants from remotely sensed data (Wang et al., 2015). Some researchers have utilized the PROSPECT model to derive foliar biochemical properties including nitrogen, protein, cellulose, and lignin (Féret et al., 2021; Wang et al., 2021; Wang et al., 2015). According to Jacquemoud et al. (1996), the retrieval of foliar nitrogen from fresh leaves was considered impossible using RTMs methods. However, Wang et al. (2015) estimated leaf protein from the spectra of fresh leaves, using the PROSPECT 5 leaf model, which combines the effects of foliar protein, cellulose, and lignin.

A limited number of studies, e.g., Féret et al. (2021), have directly utilized the radiative transfer model in estimating foliar *C*:*N*. According to Féret et al. (2021), until recently, the shortcomings of physically based leaf radiative models included the inability to decompose spectral components correctly and estimate nitrogen-based proteins and other carbon contents of fresh and dried leaves based on optical properties. Building on this shortcoming, they developed the PROSPECT PRO model, the most recent version of the PROSPECT model (Jacquemoud et al., 1996), that separates leaf dry mass per unit leaf area (LMA) into nitrogen-based constituents (protein) and carbon-based constituents (*CBC*). For instance, Berger et al. (2020) noted that the PROSPECT PRO model was calibrated using fresh and dry leaves and it is based on the principle that the nitrogen constituents and *CBC* (cellulose, lignin, hemicellulose, and starch) are a complementary part of the total leaf LMA. Fresh and dry leaves were used to calibrate PROSPECT PRO, and in both types of leaves, the model was validated with similar estimates of protein content. In a similar study, Féret et al. (2021) used the new PROSPECT PRO model to determine the nitrogen-based constituents of leaf protein and other carbon-based constituents using dry and fresh broadleaf and grass samples from the LOPEX dataset. They reported that the PROSPECT-PRO can determine the carbon-to-nitrogen ratio with R^2^ of 0.87 for fresh leaves, and R^2^ of 0.65 for dry leaves, based on the *CBC-*to-protein ratio. The study concluded that optimal selection of spectral features improved the assessment of leaf constituents from fresh samples. As a result, the PROSPECT-PRO model appears to be well suited for the quantification of the *C*:*N* ratios, which may be relevant for vegetation studies in conjunction with data from current and upcoming satellite sensors.

The radiative transfer model offers the advantage of robustness and transferability to other regions because its analysis of vegetation properties is established on the physical laws and it is not dependent on the sensor, site, or season (Berger et al., 2018). However, it is seldom used due to model complexity and computational challenges (Lu & He, 2019) and may be unsuitable for real-time analysis. Consequently, in recent times, the integration of the RTM such as PROSAIL with machine learning algorithms known as the Hybrid model has been used to derive biochemical and biophysical properties for vegetation (Danner et al., 2021). Literature notes that this is a promising approach to retrieving biochemical and biophysical information from earth observation sensors, for example, Sentinel 2 and Landsat 8 (Doktor et al., 2014; Rivera-Caicedo et al., 2017; Verrelst et al., 2019). For instance, Danner et al. (2021) combined the PROSAIL model with several machine learning algorithms (RF, ANN, and gaussian processing regression (GP)) to provide spatial information about foliar biophysical and biochemical properties (i.e., chlorophyll level, leaf area index, leaf mass per area) with relative error scores less than 10%. Imaging spectroscopy can be processed more quickly (Verrelst et al., 2019) because the hybrid model combines the adaptability and computational efficiency of machine learning with the physical basis of the RTMs (Berger et al., 2020).

These studies have demonstrated that recent advancements in the hybrid method have the potential to improve model accuracy in estimating plants’ biophysical and biochemical properties, whereas the robustness of the hybrid method is documented in vegetation studies (Brown et al., 2019). In Africa, within a semi-arid landscape, Kganyago et al. (2020) noted low accuracies in the leaf area index with RMSE > 1 m^2^ m^−2^. Therefore, it is necessary to test the accuracy and reliability of hybrid models compared to RTM as a more viable option in estimating rangeland biochemical concentrations such as *C*:*N* ratio

## Factors affecting the spectral characteristics of foliar nutrients

The spectral absorption bands sensitive to foliar biochemical such as C and N dominate the short wave infrared (SWIR) and near-infrared (NIR) region (Curran, 1989; Kokaly & Clark, 1999; Ramoelo et al., 2011). In the SWIR, the absorption features for leaf biochemical concentration include cellulose, protein, starch, and lignin. Also, specific wavebands for C have not been identified; however, the SWIR has been reported to predict foliar C concentration (Benseghir & Bachari, 2021; Gao et al., 2020). The estimation of foliar biochemicals from dried leaves, fresh leaves, and canopies using remotely sensed data is affected by several challenges. Among these is the absorption of water in the shortwave infrared which masks subtle foliar biochemical concentrations (Clevers, 1999; Kokaly & Clark, 1999). As such, the accuracy of estimating foliar C and N is strongly affected by the absorption of water in the NIR and SWIR region, which can disguise the absorption effect of other nutrients (Curran et al., 1992). Furthermore, Asner et al. (2000) and Cho and Skidmore (2006) noted that differences in leaf traits, soil background, with atmospheric effect complicate the estimation of foliar biochemicals in the field. Several techniques such as continuum removal method, derivative spectra, and log-transformed spectra have been introduced to enhance the identification of biochemical absorption features in addition to data redundancy (Kokaly, 2001; Mutanga & Skidmore, 2003). For example, Gao et al. (2020) enhanced the detection of important bands that significantly quantify the *C*:*N* ratio in forage by using continuum-removed and first derivative spectra. This study deduced that the spectra transformation methods enhance the absorption and reflection values, eliminated noise, and increased the number of bands that can accurately predict forage *C*:*N* ratio.

The absorption of foliar biochemicals such as cellulose, lignin, and starch in fresh leaves is weak and usually masked by water. Gao and Goetz (1994) successfully developed a non-linear technique that removes the effects of water absorption from fresh leaves. The method was improved by Schlerf et al. (2010) and used to model nitrogen levels in Norwegian spruce needles.

In addition, Skidmore et al. (2010) reported that the use of remotely sensed data to determine foliar nutrients can be challenging due to the difficulty to separate the signal of biomass and foliar nutrients, especially N. This effect can be minimized at the peak of biomass, where there is high absorption in the red edge and scattering in the near-infrared region of grass spectra. During the peak of biomass, the interaction between biomass and vegetation indices such as NDVI asymptotically saturates at a certain biomass density (Mutanga & Skidmore, 2004b; Thenkabail et al., 2000). Due to the variation in grass biomass, the accuracy of estimating foliar N using vegetation indices can be compromised. Nevertheless, Mutanga and Skidmore (2004b) used narrow-band vegetation indices for biomass estimation in dense vegetation with high accuracy.

## Challenges and opportunities in estimating foliar *C*:*N* ratio

A review of literature shows that hyperspectral sensors have been used extensively to predict foliar *C*:*N* ratio due to their ability to map spatial variation and identify highly discrete spectral features in vegetation that are ignored by broadband sensors (Clevers & Kooistra, 2011). Hyperspectral sensors can provide sufficient spectral information; however, they are costly, have small swath widths, and are not readily available in developing countries. The broad band multispectral sensors may not be appropriate in the estimation of *C*:*N* ratio, as a result of the limited number of absorption wavelengths in the SWIR region. Durante et al. (2014) and Gao et al. (2020) demonstrated in different studies that bands sensitive to *C*:*N* ratio in vegetation are in the SWIR region. Advanced multispectral sensors (Rapid eye, Sentinel 2, and Worldview3) with an improved spatial and temporal resolution with specialized red edge bands have proved invaluable in vegetation studies (İleri & Koç, 2022; Sibanda et al., 2017; Vasudeva et al., 2021). There is a paucity of information on the use of these sensors to predict *C*:*N* ratio, as the red and red edge region may improve the accuracy of *C*:*N* ratio prediction in rangelands.

The use of unmanned aerial vehicles in estimating foliar biochemical ratios in rangelands remains largely unexplored. Unmanned aerial vehicles (UAV) have an edge over traditional field surveys due to their flexibility, small size, cost, and application in any chosen sites (Cerro et al., 2021). Future research on foliar biochemicals should focus on integrated technologies for multispectral sensors and UAVs in the monitoring of rangelands based on data quality (spatial image and resolution) and accessibility (technical expertise and affordability). According to Karunaratne et al. (2020), the fusion of the spatial and spectral data in sensors has gained increasing interest as a new method of estimating forage quality and quantity. This is due to its ability to overcome the saturation problems of soil background in low biomass and saturation in dense vegetation (Mutanga & Skidmore, 2004a), as canopy reflectance is captured at the top surface.

Advances in algorithms, such as integration of linear and non-linear models, deep learning, and machine algorithms, can further improve the accuracy and identify the optimal variables using variable importance in projection techniques to determine the *C*:*N* ratio in rangelands. This prompts the need for future research on rangelands to test the strength of advanced sensors with machine learning algorithms, non-parametric, and combined machine learning algorithms with non-parametric approaches in the estimation of foliar *C*:*N* ratio. Also, factors (i.e., environmental and climatic) that influence the spatial variation of *C*:*N* ratio in rangelands remain unknown. Thus, future studies need to investigate the stoichiometric fluctuations of rangeland *C*:*N* ratio response to topo-climatic variables within the ecosystem.

The PROSPECT-PRO inversion model has provided a successful approach to estimating the *C*:*N* ratio using the *CBC*: proteins (Féret et al., 2021), but its use is still limited by several challenges. For instance, they reported that discrepancies in sample numbers might lead to errors and uncertainties in the calibration and validation datasets. To address this challenge, it is suggested that additional free datasets with accurate VNIR and SWIR spectral properties with commensurate detailed and reliable laboratory analysis of foliar biochemical composition are required. Furthermore, despite the shortcomings of chlorophyll-a +b contents as indicators for the estimation of nitrogen (Homolova et al., 2013), it has the advantage over protein due to its high signal at the VNIR (particularly red edge), enabling more accurate estimations even at canopy levels (Malenovský et al., 2013). It is therefore likely that a systematic and rational combination of chlorophyll monitoring with PROSPECT PRO would provide an enhanced assessment of the *C*:*N* ratio in vegetation and periodic fluctuations by separating the *CBC* and protein contents. Hence, further studies should focus on the use of RTMs in the estimation of *C*:*N* ratio in rangelands. The use of the PROSPECT-PRO model may prove invaluable in the estimation of *C*:*N* ratio in rangelands.

## Conclusion

This review has provided an overview of remote sensing techniques utilized in the estimation of the *C*:*N* ratio in rangelands with associated challenges and opportunities. Grassland *C*:*N* ratios are indicators of nutrient utilization and limitation within rangelands, and they vary in different locations. Majority of the studies on foliar *C*:*N* ratio have mostly focused on forest and croplands, while less effort has been exerted on rangelands. Although the conventional methods have been reliable, remote sensing is a non-destructive, rapid, and cheaper means of estimating foliar nutrients in rangelands at a landscape scale. Remote sensing offers spatially explicit and periodic information on the available nutrients over regional and inaccessible areas. Also, most studies have used hyperspectral data in determining foliar *C*:*N* ratio. Despite the success of hyperspectral data in estimating foliar *C*:*N* ratio in plants, they are costly and not readily available, especially in resource-scarce and financially constrained countries in Africa. Further, this study reveals that the estimation of foliar *C*:*N* ratio in rangelands, using freely available high-resolution multispectral, such as Sentinel 2 and Landsat 8, is still in infancy. The success of Sentinel 2 is attributed to the red-edge bands; therefore, more studies should test the red-edge bands in estimating *C*:*N* ratio, particularly in rangelands. In addition, literature shows that integrating remotely sensed data with ancillary data (topographic and climatic) can improve the accuracy of estimating foliar biochemical ratios. Hence, the application of integrated multisource data needs further investigation in mapping *C*:*N* ratio, especially in rangelands. The successful estimation of *C*:*N* ratio depends on the availability of robust algorithms that can improve model accuracy, flexibility, and reduce multicollinearity problems. This information is important to rangeland managers on reliable, up-to-date data in identifying the limiting nutrients and grazing patterns for effective management of rangelands. This provides a better understanding of population dynamics at various spatial and temporal scales.

## Data Availability

Data sharing is not applicable to this article as no datasets were generated or analyzed during the current study.
